# Supplementary material for: Interindividual variability in the benefits of personal sound amplification products on speech perception in noise: A randomized cross-over clinical trial
Source: PLoS One. 2023 Jul 19;18(7):e0288434. doi: 10.1371/journal.pone.0288434 (PMC10355429; doi:10.1371/journal.pone.0288434)
Supplement: S2 File — (DOCX) [file pone.0288434.s002.docx]

**Background Information**

Hearing loss is the third most common chronic disability among Canadian seniors. Mild to moderate hearing loss in older adults is associated with communication deficits, including difficulty following a conversation in a noisy environment. It is also associated with accelerated cognitive decline and the early onset of dementia. In a recent study by our team (Perron et al., submitted), we showed that older adults who reported hearing problems had poorer executive functions than those who reported few hearing problems, suggesting a strong link between cognition and hearing.

Yet, despite its adverse effects on communication skills and cognition, mild to moderate hearing loss remains largely untreated, with only 20% of older adults with a hearing loss having access to hearing aids. In most Canadian provinces, Health Insurance covers only a portion of the cost of hearing aids, which can be thousands of dollars, making them unaffordable for many older adults.

A promising and much cheaper alternative is personal sound amplification products (PSAPs), electronic, portable, over-the-counter devices amplifying sound. They are analogues to drugstore reading glasses and do not require a prescription or hearing evaluation. PSAPs are becoming increasingly popular as millions of people in the United States use these devices to compensate for their hearing loss.

However, it remains an open question whether PSAPs can effectively reduce the adverse effects of hearing loss. As these devices gain popularity, there is an urgent need to objectively evaluate the impact of PSAPs on auditory processing and communication skills.

**Research Question and Hypothesis**

This research investigates the immediate neurobiological and behavioural effects of PSAP use on speech-in-noise performance. The specific objectives are to:

1. Clarify whether using PSAPs is associated with improved speech perception in noise compared to an unaided condition in individuals with normal hearing or mild to moderate hearing loss.
2. Determine the role of listener characteristics, such as age, sex, education, self-reported hearing loss, objective hearing loss, and cognitive status, on performance during PSAPs use.
3. Study the neurobiological mechanisms of benefit using electroencephalography (EEG).

Our hypotheses are twofold:

1. We expect that wearing PSAPs for a single session will improve performance and lower self-reported listening effort during speech-in-noise perception.
2. We expect that the behavioural benefits of PSAPs will be associated with increased brain activity in auditory areas and increased functional connectivity between auditory and speech regions.

**Method**

**Participants:** A total of 32 participants will be recruited. This sample size is similar to or larger than other studies examining the impact of PSAPs. Participants will be adults between the ages of 60 and 90 with normal hearing or mild to moderate hearing loss (no clinical diagnosis) who have never used hearing aids or any other type of hearing device.

Participants will be recruited through the Rotman Research Institute participant database. The selection of participants from the database will be based primarily on the hearing test results already available in the database. If necessary, additional participants will be recruited through advertisements.

Participation is voluntary and anonymous. All participants will sign a consent form before the start of the study and receive a modest stipend of $15 per hour for their time. Reimbursement for parking fees or RTC tickets will also be provided.

**Inclusion criteria:**

- Be between 60 and 90 years old.
- Normal hearing or mild to moderate hearing loss (i.e., hearing thresholds between 25 and 60 dB in both ears).

**Exclusion criteria:**

- Left-handed and ambidextrous (this is important to know given that handiness has been shown to influence speech perception);
- Mother tongue not English (this is also important because of differences in speech perception between monolingual and bilingual individuals);
- Language impairment (because this could be confounded with hearing impairment);
- Dementia (as it can affect the understanding of task instructions and the ease of fitting devices);
- Cerebrovascular diseases (as we study brain activity, and that can affect brain activity);
- Untreated vision impairment (as it can affect the reading of task instructions);
- Tinnitus and otologic disorders (as they can affect hearing);
- Cochlear implants and hearing aids (as these devices are used to treat hearing loss);
- Diagnosed addiction (alcohol or drugs) (as we study brain activity, and that can affect brain activity);
- Significant medical or neurocognitive conditions (other than MCI) or interventions likely to significantly impact cognitive function (e.g., epilepsy, stroke, traumatic brain injury with loss of consciousness > 5 minutes, brain tumour, multiple sclerosis, hepatitis C, developmental delay, electroconvulsive therapy) (as we study brain activity, and that can affect brain activity);
- DSM-V diagnosis of major depressive disorder with active symptoms within 90 days of study entry, past or present psychosis, or other psychiatric disorders such as obsessive-compulsive disorder, generalized anxiety disorder, and bipolar disorder (as we study brain activity, and that can affect brain activity).

**Study Protocol:**

This study will consist of two sessions of three hours separated by one week apart. This study will include hearing evaluations, auditory and cognitive tasks, and various questionnaires assessing health, anxiety, depression, and listening ability.

In one session, participants will perform the auditory tasks without using any hearing devices, and in the other session, participants will be fitted, under laboratory supervision, with bilateral PSAPs. The order of the two sessions will be counterbalanced across participants so that some participants will complete the tasks with PSAPs in the first session and others in the second session. Counterbalancing will be generated by randomly assigning an order to each participant using the block randomization method (three blocks).

In each session, participants will perform the same tasks. Cognitive assessment and pure-tone audiometry will be conducted only in the first session.

In each session, the participant’s brain activity during the speech perception tasks will be recorded by electroencephalography (EEG) using a 76-channel Active Two acquisition system (BioSemi V.O.F., Amsterdam, The Netherlands).

**Clinical Trial Registration:**

Theoretically, this study meets the definition of a clinical study since we plan to evaluate the effects of a device on health outcomes. However, participation in this study involves fewer obligations than formal clinical studies. Participants will only wear the devices for one three-hour session, and no medication is applied.

This clinical trial is registered here: https://www.clinicaltrials.gov/ct2/show/NCT05076045/.

**EEG Acquisition:**

During each session, participants will be fitted with a cap containing 76 specially designed sensors to monitor brain activity using an electroencephalogram. Sensors will also be placed on the cheeks and forehead. To record a good brain activity signal, we will need to abrade (i.e., scrape) a bit of the scalp at each sensor location. Then, the gel will be inserted into each sensor. Brain activity will be recorded during the speech perception task. The preparation for the EEG is about 1 hour.

Participants may develop a headache or note some mild tenderness in the scalp area due to the placement of the brain-activity sensors. This occurs infrequently and is short-lasting.

**Telephone Interview Questionnaires (≈ 45 minutes):**

1. Demographic and Health Questionnaire: This questionnaire will document demographic and health information. Information on age, gender, education level, country of birth, race, ethnicity, language, and health will be documented. This questionnaire will be completed with the participant during a telephone interview to assess eligibility. The duration of this questionnaire is approximately 30 minutes.
2. The Edinburgh Handedness Inventory: This questionnaire is a measurement scale used to assess the dominance of a person's right or left hand in everyday activities. This questionnaire will be completed with the participant during a telephone interview to determine eligibility. The duration of this questionnaire is approximately 5 minutes.

**In-person Questionnaires (≈ 20 minutes):**

1. Speech, Spatial and Qualities of Hearing Scale (SSQ): This questionnaire measures self-perception of hearing. It consists of 49 questions divided into three sections documenting hearing abilities in different daily life situations. The questionnaire includes 14 questions on speech-related hearing disabilities, 17 questions on spatial-related hearing disabilities and 18 questions on other hearing disabilities. For each question, participants will be asked to indicate on a scale of 0 to 10 if they would be completely unable (score of 0) or perfectly able (score of 10) to perform what is described in the question (e.g., understand speech in noise). The duration of this questionnaire is approximately 10 minutes.
2. The 15-item Geriatric Depression Scale (GDS): This questionnaire is designed to detect symptoms of depression but is not used for diagnostic purposes. It consists of 15 yes/no questions about mood, aspirations, and daily activities. The duration of this questionnaire is approximately 5 minutes.
3. The 10-item Geriatric Anxiety Scale (GAS): This questionnaire is designed to detect anxiety symptoms but is not used for diagnostic purposes. It consists of 10 questions that are answered with “Not at all,” “Sometimes,” “Most of the time,” and “All of the time.” The questions also concern mood, aspirations, and daily activities, focusing on anxiety. The duration of this questionnaire is approximately 5 minutes.

**Cognitive Assessment (≈ 20 minutes):**

1. Montreal Cognitive Assessment: General cognitive functioning will be assessed using the Montreal Cognitive Assessment Hearing Impaired (MoCA-HI), a 10-minute cognitive tool. This assessment measures several cognitive domains, including orientation, memory, visuospatial function, executive function, and language.

**Hearing Tests (≈ 15 minutes):**

1. Pure tone Audiometry: Pure tone audiometry will measure hearing. During this test, pure tones are sent through headphones into one ear and then into the other. This allows us to determine the lowest intensity at which the sounds are perceived. Pure tone thresholds will be measured separately for both ears at the following frequencies: 250, 500, 1000, 2000, 3000, 4000, 6000, and 8000 Hz. This test will take about 10 minutes.
2. Quick Speech in Noise (QuickSIN) test: The QuickSIN test estimates speech-in-noise ability. Four sentences with five keywords per sentence are presented in four-talker babble noise. The sentences are presented at pre-recorded SNR ratios which decrease in 5-dB steps from 25 (very easy) to 0 (very difficult). The duration of this task is approximately 5 minutes.

**Auditory Tasks (with EEG recording) (≈ 2 hours, including EEG preparation):**

1. Syllable discrimination task: The task is to discriminate pairs of syllables as identical or different in three intensities of background noise. The duration of this task is approximately 10 minutes. Three hundred syllable pairs will be presented, including 150 identical and 150 different pairs. The task will be divided into three sections. For each section, participants will be asked to estimate the effort required to understand the words in the presence of background noise using a scale ranging from "no effort" to "extreme effort." Participants will be asked to respond using two keys on a computer keyboard.

**Statistical Analyses:**

The outcome of the QuickSIN is the signal-to-noise ratio (SNR) loss. Analyses will depend on the normality of the scores. Normal: Paired-sample t-test; non-normal: Wilcoxon signed rank test.

The outcome measures for the syllable discrimination paradigm are reaction time and accuracy. We will use linear mixed-effects (LME) models separately for each dependent variable. Models will include Session (without, with) and SNR as within-subject factors. For self-reported listening effort, the listening effort score will be analyzed using a cumulative link mixed model.

Regression models with health and demographic factors will be conducted to identify predictors of the benefits.

Brain activity will be recorded using EEG. EEG recordings will be processed offline using Brain Electrical Source Analysis software (BESA 7.1). Measures of sensory-evoked response, event-related potential and connectivity will be analyzed using BESA Statistics.

**Confidentiality:**

The research staff will collect and record in a research file only the information necessary for the proper conduct of the study. This information will remain strictly confidential and be kept under lock and key in the researcher's laboratory in charge of the research project. The data will be held for ten years. If participants withdraw their consent, the data will be destroyed immediately. To ensure confidentiality, the following measures will be provided:

- The name(s) and surname(s) of the participants will not appear on any report;
- Participants’ names will be replaced by a code when signing the consent form. This code will be used on all research documents. Only research staff directly involved in the project will have access to the list of names and codes;
- If the information obtained in this research is to be analyzed later, only the code will appear on the documents;
- Under no circumstances will individual results be disclosed to anyone without the participants’ consent.

**Data Sharing:**

Participant data, such as behavioural measures and EEG recordings, will be shared on the Open Neuro platform (https://openneuro.org) or Scholars Portal Dataverse (https://dataverse.scholarsportal.info). Shared data will be further anonymized by replacing the participant code with a generic code. Participants will be informed of this sharing in the consent form. Participants will be able to withdraw their data from sharing if desired. Withdrawal from data sharing will not affect participation in the study.

A summary of the results will also be published on <https://www.clinicaltrials.gov>. The participants will be informed in the consent form.

**Significance/Impact**

The results will also provide important new information on the effectiveness of PSAPs in addressing communication deficits of older adults with normal hearing and mild to moderate hearing loss. This will help provide guidelines for future low-cost device-based interventions for people who cannot afford conventional hearing aids and even more so for those at risk for cognitive impairment.
