# Supplementary material for: Interindividual variability in the benefits of personal sound amplification products on speech perception in noise: A randomized cross-over clinical trial
Source: PLoS One. 2023 Jul 19;18(7):e0288434. doi: 10.1371/journal.pone.0288434 (PMC10355429; doi:10.1371/journal.pone.0288434)
Supplement: Attachment — Submitted filename: Response to reviewers.docx [file pone.0288434.s003.docx]

**Response to reviewers**

**Reviewer #1**

Major concerns

1. However, 13 of the 28 participants had normal hearing. It is unclear to this reviewer why participants with normal hearing were included given the rationale for the study related to mitigating hearing loss with low cost PSAPS. I suggest removing the normal hearing participants as I have difficulty understanding the interest in studying this group. If they are to be included, consider adding Group as a between subject variable in the statistical analyses to determine if the effects—although small—are different for normal versus impaired groups. By including the normal hearing participants in the current analysis, I suspect that the statistical power has been increased to the point where these small effects reach statistical significance.

Thank you for your comments on including participants with normal hearing in our study. While we recognize that the primary objective of our study was to investigate the effectiveness of PSAPs in alleviating hearing loss, we decided to include participants with normal hearing for two reasons. First, research has shown that even people with normal hearing can have difficulty listening to speech in noise. PSAPs could therefore help them communicate better through noise cancellation and amplification features. Second, PSAPs are devices designed for people without hearing loss who need amplification in certain situations, and by including participants with different hearing profiles, the study can investigate the true extent of the devices' effects. The sample size for the hearing groups (13 participants with normal hearing and 15 participants with hearing loss) might be too small, and conducting separate analyses for each group might not provide sufficient statistical power to detect significant differences. Therefore, we decided to analyze data from both groups together to increase the statistical power of our study.

In response to reviewer #3's suggestion, we performed new statistical analyses that included the interaction between demographic factors and session as fixed effects. This approach allowed us to examine the effects of hearing loss in a more nuanced way, given that hearing thresholds are more of a continuous variable than a dichotomous one.

Our analyses revealed that hearing loss has a complex effect on task performance. Specifically, we found that hearing loss predicted performance only on the QuickSIN. These results suggest that PSAPs benefit a wide range of individuals, including those with mild hearing loss and those with normal hearing. Our results support the inclusion of participants with different hearing profiles in the study and underscore the importance of studying the effects of PSAPs on a wide range of individuals.

In response to your comment, we have revised the study objective to clarify the rationale for including participants with different hearing profiles on page 7 (lines 121-130) and acknowledged the limitations of our small sample size in the manuscript on page 32 (lines 660-665). The new analyses are detailed on pages 18-19 (lines 361-368) and the results can be found on pages 22-23 (lines 442-463). We hope these explanations and changes address your concern.

2. Many studies investigating PSAPs and OTCs note that these devices are self-fitted by the individual, yet they typically involve assistance from the research team. This is a flaw of the current study as well. Page 11, lines 215-227, detail the fitting process of the device. Lines 220-221 suggest the participants were not guided or assisted and this fitting was typical of anyone that would use the device. The lines above suggest otherwise as these participants were fitted in a double-walled sound booth, earmolds were selected by the researchers, instructions and examples regarding the fitting were provided, additional assistance was provided if requested. It is unclear why the researchers did not provide the device and instructions as any person that purchased these devices on-line would receive, have them do an actual self-fit, then do the testing.

We have modified the section on fitting to reflect the process better. The process was primarily done by the participants themselves. Specifically, they were responsible for selecting the ear tips that were most suitable for them and performing the fitting test in the app. Instructions regarding the hearing tests were given via the app.

The experimenters provided little guidance unless absolutely necessary. We did, however, help participants confirm that the devices had some degree of noticeable amplification for them and that they were functioning properly, as we were interested in the effects of the devices, not the participants' ability to use them. We have clarified the process on pages 10-11 (lines 197-222).

We agree that given the popularity of the devices and the ease with which they can be purchased, additional studies are needed to verify that participants can self-fit the devices and that self-fitting provides benefits.

Other concerns:

3. Page 8, participants, please include a power analysis to determine the appropriate sample size.

We agree that power analysis is an important component in planning a study and determining an appropriate sample size. Power analysis is crucial in ensuring that the study has sufficient statistical power to detect meaningful effects and to minimize the risk of a type II error. However, we encountered a challenge in conducting a power analysis for our study as previous studies on PSAPs did not report effect sizes or descriptive statistics of group/condition differences. As a result, we based our choice of sample size on theirs, as we believed that using the same sample size would provide comparable effects.

We have acknowledged this limitation in the manuscript page 32 (lines 660-665) and added a statement in the methods section on page 8 (lines 141-143) to clarify the choice of sample size.

In the current manuscript, we have provided effect sizes for all analyses, which will help to address this issue. These additions will provide a better understanding of the power of our study and its ability to detect meaningful effects.

4. Figure 1 is not necessary.

We understand that the information presented in Figure 1 is also detailed in the text and may be redundant. However, we would like to emphasize that Figure 1 is mandatory for randomized clinical trial reporting according to the CONSORT guidelines. As our study falls under the category of a clinical trial, we included this figure to adhere to the guidelines. We hope this explanation clarifies the rationale for including Figure 1 in our manuscript.

5. Page 10, line 204-205, the rationale for using this device stated here is vague. What is this based on—‘good representation’? I suggest deleting this sentence. The sentence that follows makes a stronger rationale for selecting this device.

We acknowledge that the sentence in question may have been vague and have removed it from the manuscript (page 10, lines 198-199).

6. Page 11, lines 208-209, is this $300 cost per device or for 2 devices? Also, I searched this device on-line and it appears to be a BTE and not an in-ear device. Thank you for bringing up this point.

The cost of $300 is per device. We apologize for the confusion regarding the type of device used in our study. The PSAPs used in our study were behind-the-ear (BTE) devices. We have updated the manuscript on page 10 (lines 202-203) to reflect this information.

7. Page 11, line 210-211, consider changing this description to ‘dual directional microphone that is more sensitive to sounds from the front and less sensitive to sounds from the back.’

The change has been done on page 10 (lines 201-202).

8. Page 11, line 224, was the NAL-NL2 fitting used based on the app? Is this the only option available, or was this an option selected by the researchers for the participants?

We revised the entire fitting process section (pages 10-11, lines 197-222). to increase reproducibility and provided more detail on the specific fitting procedures used in the study.

After the hearing test in PSAPs, the mobile app automatically determines the level of amplification required by the participant. When we contacted the company for more information, they told us that the amplification is calculated based on the NAL-NL2 procedure. This is the only option available. Since the application is a commercial application, we could not access any other information.

9. Page 12, line 251, McArdle & Wilson (2006) determined the QuickSIN lists were not equivalent in difficulty for listeners with hearing loss and suggested lists 4, 5, 13 & 16 should not be used. List 4 was used in the current study.

Thank you for bringing up the study by McArdle & Wilson (2006) to our attention. However, we would like to note that since the order of the lists was counterbalanced across participants and sessions in our study, it is less likely to have influenced our results. Additionally, we re-ran the analyses without including list 4 and still identified a significant effect of PSAPs on the QuickSIN, *p* = 0.015, *r* = 0.46. Therefore, we are confident that our results are not solely attributable to list 4.

10. Page 13, line 261, the example for identical is /tap/-/bat/ but these words are different.

Correction has been made on page 12 (line 251).

11. Page 13, line 275, were the words presented through 2 speakers simultaneously? Why would you present the words from 2 speakers at +/-45 degrees as this isn’t a realistic situation.

This design was intended to mimic a complex and immersive listening environment. This has been explained on page 13 (lines 265-270). While it is correct that the sound source of a single speaker must be localized to a specific location, the background noise is generally more diffuse. To minimize the differences between the front and back signals, we presented the stimuli at a 45-degree angle.

12. Page 14, lines 290-293, why did you change from referring to the stimuli as CVC words to ‘syllables’?

We agree that confusion is caused by using the terms 'syllables' and 'words'. To clarify, the stimuli used in the study were monosyllabic words, consisting of only one syllable. We understand how referring to the stimuli as words may increase clarity, and we have modified our terminology accordingly.

13. Page 15, line 311, is Field a typo?

“Field” is a typo. The correction has been made on page 14.

14. Page 20, line 398, why is there no result reported for the interaction of SNR x session?

We omitted the interaction because it was not significant. We have clarified the results section by presenting several statistics in table form. The interaction between Session and SNR is now presented in Table 3.

15. Page 24, lines 503-505, I am not sure the data support this statement, particularly the notion this could reduce the negative impact on cognitive performance. Also, a picky thought, but hearing aids do not restore hearing. Same on page 28, line 593.

We have revised the wording on page 25 (lines 490-497) to reflect the results of the study more accurately. Additionally, we have changed the term 'hearing restoration' to 'hearing amplification' to reflect a more accurate terminology.

16. Lastly, the manuscript is lengthy and has quite a bit of redundancies throughout. I get frustrated when a reviewer makes a comment that doesn't have a direct solution, so I realize this isn’t helpful. Just consider.

We have taken this comment into account in revising the paper. The new tables, the new analyses, and the clarification of the methodology improve the clarity of the paper. We have additionally removed some redundant information throughout the manuscript.

**Reviewer #2**

1. The following are to be provided/stated/described:
   Line 154, the word abbreviation sd
   Line 161, exclusion criteria
   Line 168, the condition
   Line 172, the second session.
   Line 181, allocation concealment.

Line 179-182, information to be incorporated into Figure 1.

We are pleased to inform you that we have made the changes you suggested and modified the participants section to provide clearer information on page 8 (lines 148-154) including the exclusion and inclusion criteria and the concealment allowance. We hope that these modifications address your concerns.

Regarding your suggestion to revise Figure 1, we would like to clarify that the current version is based on the CONSORT guidelines. The CONSORT guidelines only include information regarding the number of participants in each study step.

2. The study design not very clear and requires more description and with proper flow.

We have updated the study design on page 9 (lines 161-179) for clarity and more information. We hope these changes will improve the flow.

3. Line 208, $300 US to be written as USD300

We have made the necessary changes on page 10, line 202.

4. Line 269, ‘((SNR; Pressuresignal/Pressurenoise) were used (SNR +3 dB, SNR 0 dB, SNR -3 dB).’ to be rewritten.

This has also been changed on page 13, lines 259-260.

5. Line 311, typographical (Field[27].

We have removed the term 'Field.'

6. Line 319, “very good,” “good,” “moderate,” “bad,” or “very bad” is to be presented as 'very good', 'good', 'moderate', 'bad' or 'very bad'.

We have made the changes on page 15 (lines 304) and presented the response options as suggested.

7. Line 325, the title name is too short.

We have revised the title to provide a more descriptive and informative title. The title can now be read as follows: “Table 1. Demographic and Health Characteristics of Study Participants (n = 28)”. Please see page 16 for the updated table.

8. Line 341, the level of the accepted statistically significant to be stated.

We have now included the level of accepted statistical significance in the analyses section on page 17 (lines 335-336). Specifically, we used a p-value threshold of 0.05 to determine statistical significance.

9. Table 1, n to be stated. In the footnote, ‘with’ and ‘without’ to be denoted.

We have updated Table 1 to include the sample size (n) in the title and added a footnote to denote the meaning of "with" and "without". Please see page 16 for the updated table.

10. Range to be denoted as minimum to maximum.

Additionally, we have revised the range to be denoted as "minimum to maximum." Please see page 16 for the updated Table 1

11. Some results presented in the text are best presented in table form.

Descriptive statistics for changes in performance with and without the devices, as well as inferential statistics for the linear mixed models of the syllable discrimination task are not presented in Tables 2 (page 19) and 3 (page 21), respectively.

12. Line 367, the fulfillment of multiple stepwise regression analyses assumptions to be stated. Whether interaction and multicollinearity were explored is to be stated.

Based on Reviewer 3's suggestion, stepwise multiple regression analyses were replaced with linear mixed models. We have updated the manuscript to present these changes.

Based on your comment, we have clarified that we have checked the analysis assumptions for all analyses (page 18, lines 361-368).

13. Line 469-470, the sentence to be rewritten.

The sentence has been rewritten on page 28, lines 563-565.

14. List of references to conform to the journal format.

The current reference list is formatted with the PLoS Endnote style that can be found here: <https://endnote.com/style_download/plos-public-library-of-science-all-journals/>

**Reviewer #3**

1. One major concern is the conceptual differences between the PSAP devices tested here and the over-the-counter hearing aids. The title and various other parts of the paper describe PSAP and OTC devices interchangeably, but a more careful description of how the devices used in this study compare to OTC devices would be helpful. There is some overlap between these terms, but they are also distinctions that should be made so that readers understand the type of device that was evaluated and how that might compare to the more general category of OTC devices. Referencing the US FDA Final Rule on OTC definitions might help provide additional detail on how to classify this device using terminology that has recently become familiar to readers.

Thank you for this important comment! We updated the introduction and discussion section to explain the conceptual differences between conventional hearing aids, OTC hearing aids and PSAPs. Changes can be found in the introduction on page 5 (lines 74-91) and in the discussion on pages 26-27 (lines 528-539). We have also carefully reviewed the manuscript to ensure that there is no confusion in the terms used.

2. The description of the process of fitting the devices was confusing and replicating this study based on the details provided in the method would be challenging without further elaboration.

We revised the entire fitting process section (pages 10-11, lines 197-222) to increase reproducibility and provided more detail on the specific fitting procedures used in the study.

2.1. The is reference to an earmold in the fitting. An earmold is traditionally a custom mold fitted to the specific patient’s ear by taking an impression. A clear description of the device coupling to the ear should be made in terms of whether this was a custom fitting or if the fitting was non-custom using different sized domes or ear tips.

The reference to an earmold has been changed to indicate that ear tips were used. Three different sizes of earmolds are provided with the devices at the time of purchase. Participants were asked to choose the size that best fit their ear holes. The change can be found on page 10, lines 204-206.

2.2. It says that the patients self-fitted the device, but in one sentence (Line 218) instructions were given and in the next sentence (Line 220), it says that fitting assistance was not provided to simulate self-fitting at home. This should be clarified as it might impact how these results relate to current OTC devices that do not include professional guidance or instructions.

We have modified the section on fitting to reflect the process better. The participants themselves primarily did the process. Specifically, they were responsible for selecting the tips that were most suitable for them and performing the fitting test in the app. Instructions regarding the hearing tests were given via the app.

The experimenters provided little guidance unless necessary. We did, however, help participants confirm that the devices had some degree of noticeable amplification for them and that they were functioning properly, as we were interested in the effects of the devices, not the participants' ability to use them. We have clarified the process on pages 10-11.

2.3. The fitting procedure describes a hearing test, assumably through the device (in situ), but that is not clear.

This has also been clarified in the fitting section (pages 10-11, lines 206-208). The hearing test was conducted in the device (in situ).

2.4. The device based the fitting on the NAL-NL2 prescriptive formulae, but in the previous paragraph the device is described as having three pre-sets. Thus, it is unclear whether the fitting was customized based on the hearing test or if the device selects one of the presets closest to NAL-NL2 based on the hearing test. This is important because fitting to NAL-NL2 targets using only three presets for the range of hearing losses included in the sample would be difficult.

The reference to the three presets has been removed to avoid confusion. After the hearing test in PSAPs, the mobile app automatically determines the level of amplification required by the participant. When we contacted the company for more information, they told us that the amplification is calculated based on the NAL-NL2 procedure.

2.5. Was the fitting verified in any way using probe microphone or electroacoustic measures to determine whether the devices provided consistent audibility or how closely they matched the NAL-NL2 targets?

Unfortunately, we did not verify this using objective methods. However, we relied on participant feedback to confirm that they were noticing changes in amplification, to ensure that the devices were working properly or providing at least some degree of amplification. This has been explained on page 11, lines 215-220.

3. The lack of verification of the output of the devices is a major limitation of the experimental design. The large individual differences in benefit from these devices could be related to variation in the output of the devices in the ear canal and variation in how well they were coupled to the ear. It is true that real-ear verification or coupler measures would not be available if these devices were self-fitted at home as PSAPs are, but the same argument could be made for all of the other outcomes in the experiment, and the lack of consideration for how these devices might have restored audibility for these participants is a significant limitation in that a key factor that could have determined the outcome was omitted from the design.

We used this strategy because these devices are intended to be purchased by individuals and installed by themselves. We believe that measuring self-reported amplification by the participants allowed us to ensure that the devices provided some degree of amplification.

However, we totally agree with you. The individual differences in benefit could be explained by the lack of verification of device power. We did not consider this factor in our study design, which is a major limitation of the study. We have updated the study limitations section to mention this limitation on page 32, lines 650-659.

4. The use of a difference score in the statistical analyses, rather than creating a repeated-measures fixed effect for aided condition is problematic as the difference score ignores the variance of the unaided and aided conditions. The linear mixed effects models used here with random effects for each subject can account for the correlation between repeated measures within subject quite well, so it is unclear why the repeated measure was reduced to a difference score instead.

Thank you for this important remark. We have removed the stepwise regression models with the difference score. We have run additional sets of linear repeated effects models with the interaction between demographic/health factors and session. These new models account for the variance in both conditions. These analyses reveal similar findings, strengthening the results. The analysis section has been modified on pages 18-19 (lines 361-368) to explain the statistical models and the new results are now presented on pages 22-23 (lines 442-463). Figure 6 has been modified to illustrate the new effects.

5. The stepwise regression approach based on AIC is concerning because the model comparisons and AIC criteria are not described. One can assume the mode with the best AIC was reported, but it is unclear how much better the final models were than comparator models for both speech recognition and listening effort. Much more detail about the statistical approach would be needed to interpret these models.

Since these analyses have been removed from the current version of the manuscript, we believe that this commentary no longer applies.

6. It is unclear whether the design has adequate statistical power to assess the number of predictors that were included in the model and whether the lack of power influenced the stepwise regression outcome.

We strongly agree with you that it is important to minimize the number of predictors in the analysis to avoid overfitting. Given our small sample size, we decided to focus on a subset of predictors, namely age, hearing, cognition, and self-reported hearing measures. We focused on these predictors because the hearing aid literature shows that this variable can explain the benefits of hearing amplification. This rationale has been explained on page 18 (lines 356-360). We removed gender and education from the analyses because we did not have a specific hypothesis regarding the effects in the model. We have also acknowledged the limitation of our small sample size in the manuscript on page 32 (lines 660-665).

7. The discussion includes overly general statements about potential benefits from PSAP and OTC devices that should be heavily qualified. This is an example where a clearer description of how these PSAP devices fit within the category of OTC devices would be helpful, as it could help readers to understand how the results with these devices might generalize to the broader landscape of OTC hearing aids.

Conceptual difference between the three types of devices (conventional hearing aids, OTC hearing aids and PSAPs) is now part of the discussion section on pages 26-27 (lines 528-539).

8. A minor comment on the Introduction. The need for OTC devices in the introduction is heavily based on the cost of current prescription hearing aid being too high. It is worth acknowledging several studies that have shown that hearing aid adoption and hearing aid use are only marginally better in countries where prescription hearing aids are provided for free as part of government healthcare programs or at a significantly reduced cost. This should be acknowledged, and the revised discussion should include some consideration for how the benefit of OTC devices might compare to the benefit of prescription devices if the latter were available at low or no cost.

Thank you for another very interesting comment. We now acknowledge in the introduction that cost is not the only factor in why hearing loss is undertreated, as other factors can explain adoption rate (page 5, lines 70-73). In addition, the discussion places the results in the context that hearing aids are not always reimbursed, making PSAPs and OTC hearing aids a potential alternative for people who cannot afford prescription devices. We also explain why PSAPs might still be useful if prescription devices are available at little or no cost. The changes can be found on page 27, lines 539-546.
