# Supplementary material for: Interindividual variability in the benefits of personal sound amplification products on speech perception in noise: A randomized cross-over clinical trial
Source: PLoS One. 2023 Jul 19;18(7):e0288434. doi: 10.1371/journal.pone.0288434 (PMC10355429; doi:10.1371/journal.pone.0288434)
Supplement: Attachment — Submitted filename: Response_to_reviewer_R2.docx [file pone.0288434.s004.docx]

**Response to reviewers**

**Reviewer #1**

1. The authors added justification for including normal hearing participants in the study design. The justification is “PSAPs are designed for people that have difficulty hearing in certain situations, despite having normal hearing for their age.” They further noted “We focused on speech perception in noise because it is challenging for both older adults with and without hearing loss.” However, there is no information regarding the difficulties encountered by the normal hearing participants in the study. These were not normal hearing people with difficulties in noise based on the inclusion criteria. That being said, the normal hearing participants could have been evaluated separately from the hearing-impaired participants, as suggested, yet the authors did not do so. The effects observed were already very small. Including the normal hearing participants in the current analysis has increased the statistical power to the point where these small effects reach statistical significance. The authors suggest that using PTA as a continuous variable addressed my concern; however, the results showed session was related to PTA—further suggesting normal hearing participants aren’t candidates for these devices. Moreover, throughout the discussion, the authors seem to go back and forth between the findings being small and not clinically significant to PSAPs being a viable alternative to hearing aids.

Thank you for your feedback on our study. We appreciate your comments and have carefully considered your concerns regarding including normal-hearing participants in our analysis.

We would like to clarify that the original plan of our study was to recruit a group of older adults and measure their hearing thresholds to evaluate the effectiveness of PSAPs for individuals with different hearing profiles. In our revised manuscript, we made significant changes to deemphasize clinical terminology, given that none of the participants had received a formal diagnosis of hearing loss. The sample of participants recruited contains individuals who meet the criteria of normal hearing or mild-to-moderate hearing on the basis of pure tone audiometry performed in a research setting. We modified the introduction on page 7 to clarify the purpose of the study.

For greater transparency regarding the hearing profiles of our participants, we have included additional information in the manuscript (page 10). In particular, we have highlighted that 19 participants met the criterion for hearing loss in at least one ear, which reinforces the representation of people with hearing difficulties in our study.

In response to your concern about the effects induced by people with normal hearing, we performed additional exploratory analyses specifically for the 19 participants who met the hearing loss criterion (see results section). These analyses were designed to determine whether the observed effects remained significant when focusing solely on these individuals. The results showed that most effects remained statistically significant even within this subgroup.

We hope these modifications address your concerns and improve the overall quality of our study.

2. As noted in the previous review, many studies investigating PSAPs and OTCs note that these devices are self-fitted by the individual, yet they typically involve assistance from the research team. This remains a flaw of the current study as well. In the revision, the authors note “No guidance (except if necessary) was necessary to participants during the test, as these devices are intended to be fitted at home without assistance of a healthcare professional.” How often was assistance necessary and to what extent? Again, it is unclear why the researchers did not provide the device and instructions as any person that purchased these devices on-line would receive, have them do an actual self-fit, then do the testing.

While we agree with you that we have, to some extent, proposed assistance to fitting and that this does not replicate what individuals would experience with these devices, we would like to emphasize whether the devices offer benefits and whether participants can fit them accurately themselves are two different issues. Although our study attempted to focus on the effectiveness of the devices, we agree that further studies should test whether self-fitting is realistic.

In response to your comment, we have clarified that instructions were provided to participants via the app where the hearing test was conducted. We have also clarified in which cases guidance was given and for what reason. This guidance aimed to ensure that we were measuring the devices' effectiveness, not participants' ability to set them up. Most participants were able to fit the devices themselves. Modifications have been made on page 11.

3. The test setup playing the target speech signal from 2 speakers at 45 degrees simultaneously is not realistic. The design does not, in any way, “mimic a complex and immersive listening environment, similar to those typically experienced by people with hearing loss, such as in group conversations or crowded restaurants, where sounds can emanate from all directions.” The use of a multi-talker babble, such as the one used in the QuickSin, would have been more appropriate.

Thank you again for this important comment. We have modified the rationale for this configuration to clarify that it creates a complex immersive auditory listening condition. However, we agree with you to some extent regarding the ecological validity of the task. We now address this limitation in the limitations section on page 14.

4. In the discussion, line 543-546, again the authors are making claims about the value of PSAPs for normal hearing listeners that the data do not support as presented. The normal hearing data are not evaluated alone. The same, unfounded claim is made in the conclusions.

We have modified these two sections to minimize the differences between hearing loss and normal hearing. Instead, we have indicated that these devices are beneficial for people with different hearing profiles, but particularly for those with higher hearing thresholds. Modifications have been made on page 28 and page 34.

**Reviewer #2**

1. Line 409, typo numerator.

The typo has been corrected on page 21.

2. Line 445 – 462, the results are to be presented in table form.

The results are now presented in table form (see Table 4 on page 24).

3. For the reduction of predictors in the analysis, the model fit is to be discussed.

We attempted to incorporate all the predictors initially intended for the analysis. However, we faced difficulties with model convergence when including them all simultaneously. This situation reinforces our decision to use a subset of predictors, where we carefully selected the most relevant and meaningful ones to address our research questions. As a result, we achieved successful convergence of the models and obtained interpretable results.

**Reviewer #3**

1. The fitting procedure has been substantially clarified in the revision. However, the method section now refers to the "NAL-NL2 procedure" when describing how gain for the devices is determined. There really is not an NAL-NL2 procedure, and if there were, if would suggest that the output of the devices was verified using traditional probe microphone measures. To avoid any confusion, I would state that the software set the gain prescription based on the results of the in-situ audiometric evaluation using the NAL-NL2 prescription.

Thank you for your suggestion to clarify the fitting procedure. We have carefully considered your recommendation on page 11.

2. The term "over-the-counter devices" in the title should be changed to " a personal sound amplification product" since a single PSAP device was used, unless this device is classified as an OTC under the FDA definition and regulations.

We have revised the title to align with your suggestion.
